# *EGFR*基因多态性对EGFR-TKIs治疗晚期NSCLC疗效和预后的影响

**DOI:** 10.3779/j.issn.1009-3419.2013.03.09

**Published:** 2013-03-20

**Affiliations:** 210009 南京，南京医科大学附属肿瘤医院胸外科 Department of Thoracic Surgery, Nanjing Medical University Affiliated Cancer Hospital, Nanjing 210009, China

**Keywords:** EGFR-TKIs, 肺肿瘤, 多态性, 疗效, 预后, EGFR-TKIs, Lung neoplasms, Polymorphisms, Efficacy, Prognosis

## Abstract

表皮生长因子受体酪氨酸激酶抑制剂（epidermal growth factor receptor tyrosine kinase inhibitors, EGFR-TKIs）在晚期非小细胞肺癌患者（non-small cell lung cancer, NSCLC）中的应用越来越广泛，但患者的疗效存在明显差异。目前主要是根据*EGFR*基因突变来选择患者，然而，其检测常受到肿瘤组织来源困难、技术复杂、费用高等因素限制。因此，临床亟待寻求其他生物学标记物来预测EGFR-TKIs疗效。近来有诸多研究发现*EGFR*基因多态性与晚期NSCLC患者EGFR-TKIs疗效及预后也存在相关性，现将其研究进展作一综述。

## Abstract (en)

An increasing number of patients with advanced non-small cell lung cancer (NSCLC) have been treated with epidermal growth factor receptor tyrosine kinase inhibitors (EGFR-TKIs). However, significant differences in response to EGFR-TKIs have been shown among advanced NSCLC patients. Recently, selection of patients was mainly based on *EGFR* gene mutation detection. Nevertheless, mutation detection is often limited by tumour tissues derivation, technique complexity, high cost, and so on. It is urgent to seek other biological markers to predict efficacy of EGFR-TKIs. Many studies have founded that the *EGFR* gene polymorphisms are also associated with clinical outcome and prognosis in treatment of advanced NSCLC with EGFR-TKIs. Here, we presented a review discussing the correlation between *EGFR* gene polymorphisms and the efficacy of EGFR-TKIs in advanced NSCLC.

目前在全球范围内肺癌居男性癌症死亡的首位，居女性癌症死亡的第2位^[1]^，严重威胁着人类的健康。其中，非小细胞肺癌（non-small cell lung cancer, NSCLC）最常见约占肺癌总数的85%^[2]^，但大部分NSCLC患者就诊时已属于晚期，失去了手术治疗的机会，而传统放化疗等联合治疗的疗效已达到一个平台且毒副作用明显^[3]^。近年随着靶向药物的问世，晚期NSCLC的治疗趋于靶向化，以表皮生长因子受体酪氨酸激酶抑制剂（epidermal growth factor receptor tyrosine kinase inhibitors, EGFR-TKIs）最具代表性，但其疗效存在明显差异，有报道最常见的EGFR-TKIs吉非替尼在晚期患者中有效率为8.9%-69%^[4]^。因此，临床亟待寻求经济、简便、准确性高的生物学标记物来预测EGFR-TKIs疗效。近来有诸多研究发现*EGFR*基因多态性也可以预测患者EGFR-TKIs疗效及预后^[3]^。而且，基因多态性可以通过外周血标本直接检测，具有简便、经济等特点。因此，这方面研究也越来越受重视。本文就晚期NSCLC患者EGFR-TKIs疗效及预后与*EGFR*基因多态性的关系进行综述。

## 1 *EGFR*及其基因多态性

人的EGFR基因位于7号染色体7p12.3-p12.1，全长约200 kb，编码1, 210个氨基酸组成的相对分子量为170 kDa具有酪氨酸激酶活性的跨膜糖蛋白。EGFR广泛分布于人体多种细胞（如上皮细胞、成纤维细胞及角质细胞等），并在多种上皮肿瘤细胞中过度表达^[5]^，其在NSCLC中过表达率为19%-89%^[6]^。EGFR具有促进细胞增殖与分化、抑制凋亡、促进新生血管生成和肿瘤侵袭转移等作用，研究^[5, 7]^认为EGFR高表达的肿瘤患者虽然预后差，但对EGFR-TKIs治疗却具有较好的反应。因此，围绕EGFR进行的基础与临床研究对肺癌的治疗具有重要意义。来自美国国家癌症生物信息数据库的数据显示，*EGFR*基因上存在多个多态性位点，目前研究较多的是CA简单重复序列（CA Simple Sequence Repeat, CA-SSR）多态性和单核苷酸多态性（single nucleotide polymorphism, SNP），具体阐述如下。

### 1.1 内含子多态性

#### 1.1.1 CA-SSR1(rs45559542)

CA-SSR1是目前国内外研究最多也最清楚的一个位点，位于第1内含子且靠近下游增强子区。研究^[8]^表明CA-SSR1等位基因包含的CA重复序列数为9-26不等，并且存在种族差异性。Liu等^[9]^发现亚裔人群CA-SSR1普遍较长，以(CA)20为主，而高加索人和非裔美国人则较短以(CA)16最常见。同时，体内和体外的研究均显示EGFR的转录和表达与CA序列重复数呈负相关^[10]^。有研究^[11]^发现(CA)21与(CA)16相比，EGFR转录和表达下降80%。CA-SSR1影响EGFR-TKIs疗效的具体分子机制尚不清楚，推测可能是由于CA-SSR1通过调控EGFR的mRNA及蛋白表达水平，进而影响EGFR受体信号通路的活性。目前，国内外不同研究者对这一位点的研究结果不尽相同甚至互相矛盾。Nie等^[12]^对中国患者研究发现，短CA-SSR1（至少一条等位基因CA重复数≤16）的患者与长CA-SSR1（两条等位基因CA重复数均＞16）的患者相比，有更好的近期疗效（71.1% *vs* 44%, *P*=0.014）和更长的生存时间（20个月*vs* 11个月，*P*=0.039, 2）。然而，Ma等^[4]^的研究结果则不尽相同，虽然短CA-SSR1患者也具有更好的近期疗效（*P* < 0.001），但总生存期（overall survival, OS）和无进展生存期（progression-free survival, PFS）与长CA-SSR1患者相比，没有明显差异性（*P*=0.662, *P*=0.580）。Han等^[13]^通过多元分析发现，短CA-SSR1（两条等位基因CA重复数之和≤37）的韩国患者对吉非替尼有更好的客观反应（*P*=0.029）及更长的疾病进展时间（time-to-progression, TTP）（*P*=0.014）。但Jung等^[14]^则认为CA-SSR1多态性与近期疗效及远期生存均无关联性。而在非亚裔人群中如Liu等^[15]^在高加索患者中发现，较SL/LL（SL/LL：至少一条等位基因CA重复数>16）两种基因型患者，携带SS基因型（SS：两条等位基因CA重复数均≤16）患者的PFS延长（*P*=0.03）。Tiseo等^[16]^的研究结论与此相似，认为携带(CA)16等位基因的患者具有更长的OS（*P*=0.044）。然而Brugger^[17]^、Liu等^[18]^通过对厄洛替尼治疗患者的远期生存分析发现：患者PFS及OS的差异性与CA-SSR1多态性无关。同样，Giovannetti等^[19]^和Gregorc等^[20]^研究也得出类似结论，而且近期疗效亦无区别。

#### 1.1.2 8227G>A (rs763317)

8227G>A位于CA-SSR1下游6.9 kb处，目前尚不明确这一SNP位点的功能。Jou等^[21]^通过730例患者和730例健康人组成的病例对照研究发现8227G>A多态性与台湾人群的肺癌易感性相关（*P*=0.009），尤其是携带等位基因A的不吸烟女性患肺腺癌的风险明显增加。然而，随后Girard等^[22]^通过对涉及多个种族人群的研究则认为该多态性位点与不吸烟人群肺癌发病风险无关（*P*=0.58）。最近，Shitara等^[23]^研究了8227G>A多态性与日本NSCLC患者的关系，发现肺癌术后没有接受吉非替尼治疗患者的OS与该位点无相关性（*P*=0.175, 3），而在术后由于肿瘤复发接受吉非替尼治疗的患者中，携带GA/AA基因型患者中位OS（33.3个月）明显比GG基因型患者（20个月）延长（*P*=0.044, 8），但进一步的多元分析则认为8227G>A多态性并不能作为患者预后的独立预测因子（*P*=0.223, 2）。目前，国内外对这一多态性位点报道较少，因此需要开展更多的研究来了解其具体功能、与EGFR转录和表达的关系以及在人群中的分布情况，从而更好地理解该多态性位点对肺癌治疗的影响。

### 1.2 外显子多态性

#### 1.2.1 D994D G>A(rs2293347)

D994D G>A作为同义SNP，位于第25外显子这一功能活性区，没有引起EGFR氨基酸序列改变。虽然许多同义SNP可以通过影响mRNA稳定性、转录及剪切，从而间接引起蛋白表达、结构及功能的改变^[24]^，但D994D G>A多态性是否也是通过该机制进而影响EGFR-TKIs的疗效还有待进一步研究。目前这一SNP位点研究主要见于中国和日本，非亚裔人群尚未见此类报道。Sasaki等^[25]^对术后日本患者研究发现，D994D G>A多态性更多见于年轻（年龄 < 60岁）、淋巴结转移阳性患者（*P*=0.046, 7, *P*=0.016, 8），但与接受吉非替尼治疗后的OS无相关性（*P*=0.147, 1），该作者后来的另一项研究^[23]^也发现该位点与患者的预后无关。然而，Ma等^[4]^通过对中国汉族患者EGFR基因进行全基因SNP筛查，结果发现与GA/AA基因型患者相比，携带GG基因型患者临床获益率明显增高（71.2% *vs* 37.5%, *P*=0.004, 3），中位PFS延长（11个月*vs* 3个月，*P*=0.001, 8），但两者OS亦无统计学差异（*P*=0.176）。最近该课题组对该SNP位点做了更深入的临床研究，认为D994D G>A多态性与吉非替尼临床疗效密切相关，可能成为预测吉非替尼疗效的理想遗传标记物^[24]^。最近Zhang等^[26]^还发现在中国南方人群的患者中，携带GG基因型的患者中位OS（21个月）明显长于AA基因型患者（21个月，*P*=0.036）及GA基因型患者（15个月，*P*=0.025）。

#### 1.2.2 R497K G>A(rs142285)

又名R521K G>A，位于第13外显子497密码子处，参与编码EGFR胞外域第Ⅳ亚区，在酪氨酸激酶信号通路中起重要作用。Moria等^[27]^的体外研究提示突变的497k与野生型497R相比，酪氨酸激酶活性降低、与配体结合功能下降、对细胞生长的刺激以及对原癌基因*myc*、*fos*和*jun*的诱导减弱。目前对这一SNP位点国内外研究的结论比较一致，如Nie等^[12, 28]^对中国人的研究和Sasaki等^[29]^、Shitara等^[23]^对日本人的研究及Giovannetti等^[19]^、Liu等^[15]^对非亚裔人群的研究，他们均指出R497K G>A多态性与患者EGFR-TKIs治疗后的近期疗效或远期生存无相关性。推测这可能是由于该SNP位点位于EGFR胞外区，而EGFR-TKIs主要是通过与ATP竞争胞内段上的相应结合位点，从而抑制EGFR自身磷酸化，阻断EGFR的信号传导达到抗肿瘤目的。另外，Sasaki等^[29]^还发现在没有接受吉非替尼治疗患者中，若术后淋巴结阴性则该多态性与疾病的预后无关（*P*=0.888, 2），若淋巴结阳性则AA/GA基因型患者有更好的OS（*P*=0.007, 2）。

### 1.3 启动子多态性

启动子是转录开始的部位，参与基因的转录及调控。目前研究稍多且有功能意义的SNP位点有两个，即-216G>T（rs712829）和-191C>A（rs712830），它们均位于启动子转录起始点区。体外研究^[30]^显示（G>T）改变通过调节*EGFR*基因与基本转录因子SP1蛋白的结合，使启动子活性增加30%，进而使EGFR mRNA和蛋白表达上调。和CA-SSR1多态性一样，启动子SNP分布也存在种族差异性，与高加索人相比，这两个SNP位点在亚裔人群中较少见^[31]^，尤其是-191C>A更是罕见^[14]^，考虑其在亚裔人群中研究的意义较小，故本文只对-216G>T多态性进行探讨。Jung等^[14]^通过对韩国人研究发现，与GG基因型患者相比，携带GT基因型患者具有更好的疾病控制率（87.5% *vs* 66.7%, *P*=0.042）以及更长的PFS（16.7个月*vs* 5.1个月，*P*=0.005），但OS无统计学差异（*P*=0.729）。Shitara等^[23]^对日本患者远期生存的研究结论与此类似。在非亚裔人群，Liu等^[15]^在高加索人的研究中发现，携带T等位基因患者，有更好的近期疗效（*P*=0.042）及更长的PFS（4.1个月*vs* 2.1个月，*P*=0.005），但OS亦无差异性（*P*=0.11），其后来在厄洛替尼治疗患者的研究中也发现-216G>T不能预测预后^[17]^。然而，Giovannetti等^[19]^则认为患者的疗效和预后与该SNP位点均无相关性。目前国内尚未见此方面的报道研究。

## 2 对*EGFR*基因多态性位点进行联合分析的意义

总结国内外*EGFR*基因常见多态性位点与晚期NSCLC患者EGFR-TKIs治疗关系的研究进展，即使对同一多态性位点，研究结果也不尽相同甚至互相矛盾，造成这种现象的原因可能有：①研究人群不同，不同种族的*EGFR*基因多态性位点分布频率存在差异；②样本量太小及实验设计方法不同（如不同研究者对CA-SSR1长短的定义）；③*EGFR*基因多态性位点较多，对单个位点进行分析认识较局限。因此，对若干多态性位点进行联合分析意义可能更大，如在Liu等^[15]^的研究中，单独对-216G> T和CA-SSR1多态性进行分析两者与OS均无相关性，而对其联合分析发现，与其它基因型相比，同时携带SS和-216GT/TT基因型患者OS明显延长（*P*=0.02），同样，在Gregorc等^[20]^的研究中则发现，与其它单体型比较，未含G-216-C-191单体型的患者具有更好的临床获益率（*P*=0.011）及更长的PFS（*P*=0.040）和OS（*P*=0.015）。

## 3 结语

综上所述，*EGFR*基因多态性对晚期NSCLC患者EGFR-TKIs治疗的疗效及预后均存在一定影响，虽然目前的研究结论尚不统一，需开展大样本的前瞻性研究来进一步证实。但是，随着基因多态性的深入研究，有理由相信其必将为筛选最合适的病例进行靶向治疗开辟新途径，进而为NSCLC的个体化治疗提供理想指标。
